# Dosage-sensitive genes in evolution and disease

**DOI:** 10.1186/s12915-017-0418-y

**Published:** 2017-09-01

**Authors:** Alan M. Rice, Aoife McLysaght

**Affiliations:** Smurfit Institute of Genetics, Trinity College Dublin, University of Dublin, Dublin 2, Ireland

## Abstract

For a subset of genes in our genome a change in gene dosage, by duplication or deletion, causes a phenotypic effect. These dosage-sensitive genes may confer an advantage upon copy number change, but more typically they are associated with disease, including heart disease, cancers and neuropsychiatric disorders. This gene copy number sensitivity creates characteristic evolutionary constraints that can serve as a diagnostic to identify dosage-sensitive genes. Though the link between copy number change and disease is well-established, the mechanism of pathogenicity is usually opaque. We propose that gene expression level may provide a common basis for the pathogenic effects of many copy number variants.

## Gene dosage matters

At the evolutionary level, gene duplication is an important and common process [1]; at the population level, copy number variation is the most abundant kind of genetic variation per base-pair [2]; and at the individual level, gene expression is often noisy [3]. All of these observations add up to the conclusion that for many of our genes there is good tolerance for changes in dosage. Indeed, Sewell Wright argued that even the very phenomenon of genetic dominance is suggestive of a tolerance of gene dosage changes [4]. However, for a significant fraction of the genome alteration of gene dosage has deleterious effects. This is most plainly seen in the association of copy number variants (CNVs) with human disease, including heart disease, cancers, diabetes and neuropsychiatric disorders, among others [5–8]. This dosage sensitivity reflects the generally linear relationship between gene copy number and protein product in most cases [9, 10].

A dramatic but transient form of dosage alteration occurs during every cell cycle where there is a drastic disruption to the relative ratios of gene copy number, with early-replicating DNA regions being twice as abundant as late-replicating regions during S-phase. Dosage sensitivity would predict that this should be compensated, and indeed elaborate mechanisms exist to mitigate this imbalance—it was recently discovered that in eukaryotes histone-mediated dosage compensation mechanisms dampen expression from early-replicating loci during cell replication, thus rebalancing the gene products with those from late-replicating loci [11, 12]. By contrast, in bacteria the differential ratio of early- and late-replicating genes is used as a cell cycle signal [12]. These systems have deep differences, but in both cases we see that even a transient change in relative gene dosage has noticeable consequences.

## Dosage sensitivity

**Fig. 1.**
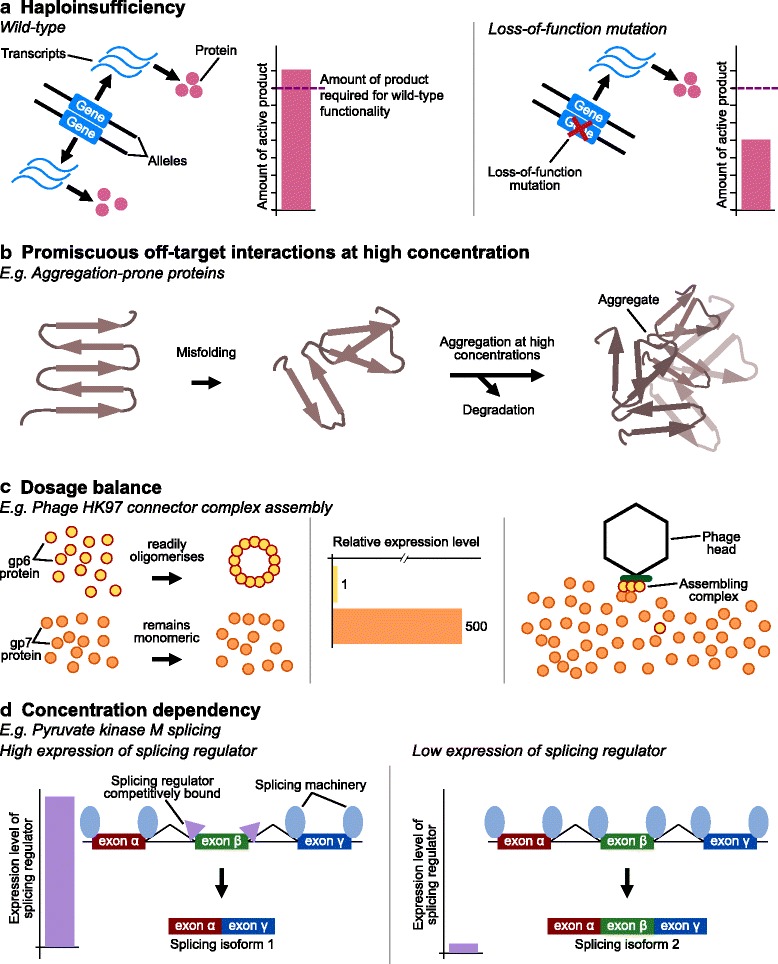
Various types of dosage sensitivity. Dosage sensitivity can be due to any of several different mechanisms. **a** For some proteins there is a minimum amount of active product required for normal function (haploinsufficiency). A hemizygous deletion or other loss of function allele will reduce the amount of active product below the threshold for functionality. **b** Some proteins form inappropriate interactions at high concentration, such as protein aggregation. These aggregates may themselves be toxic, or may phenocopy a deletion by removing the proteins from availability. **c** Dosage-balanced genes have constrained relative stoichiometry, for example the ratio of gp6 protein to gp7 protein in phage HK97 must be correct in order to achieve correct protein complex assembly. If gp6 is present in excess it preferentially forms large homomers, thus becoming unavailable to form the complex with gp7. **d** A simplified, hypothetical example of concentration-dependent activity based on the splicing of pyruvate kinase M, where the concentration of the splicing regulator determines its location of binding, which in turn determines which isoform is produced. Panel **d** is a modified from [25]

There are several different ways in which gene dosage can matter (Fig. 1). Haploinsufficiency, where a hemizygous state does not produce sufficient gene product for correct function, proposed by Wright as a source of dominant negative effects [4], is perhaps the most intuitive of the dosage constraints and has long been recognised as a cause of human disease [13], 22q11 deletion syndrome being just one well-studied example [14]. A recent large survey of human genetic variation identified over 3000 genes in the human genome with a near-total absence of loss-of-function alleles, suggesting that many of these are in fact haploinsufficient; over 70% of these were not previously associated with disease [15].

By contrast, why the presence of a surplus copy of a perfectly good gene should be deleterious is less obvious. Charcot-Marie-Tooth disease, a hereditary neuropathy, was one of the first human diseases shown to be due to duplication of a dosage-sensitive gene, *PMP22* [16, 17]. The fact that some phenotypically normal individuals carry both a duplication and a compensatory deletion of this gene supports the dosage sensitivity model rather than any other regulatory or structural effects as the underlying mechanism behind the disease condition [18]. In some cases, especially for intrinsically disordered proteins, the basis of pathogenicity may lie in an increased propensity for low-affinity off-target interactions at high concentrations [19, 20]. For example, extra copies of the α-synuclein gene (*SNCA*) are associated with early-onset Parkinson’s disease, possibly due to greater protein concentration increasing the likelihood of protein aggregation [21, 22]. Though, mechanistically speaking, why protein aggregates should have such devastating effects remains unclear [23].

Yet other genes are sensitive to both increases and decreases in copy number: many developmental morphogens act in a concentration-dependent manner [24]; whether pyruvate kinase M (*PKM*) is spliced into the adult or embryonic isoform depends on the concentration of hnRNP proteins, with high concentrations in cancer cells resulting in the ectopic production of the embryonic form [25]; and some sets of genes have constrained stoichiometry such that deviations from the normal ratio is deleterious, that is, they are dosage balanced [26]. Members of protein complexes may be particularly dosage balanced as deviations from the correct ratios of subunits can disrupt the biochemistry of protein complex assembly in non-linear ways, such that a 50% decrease in the amount of one component can result in a greater than 50% decrease in the amount of active product. Similarly, and somewhat counter-intuitively, even an increase in one of the components can result in a decrease in the amount of complete protein complex produced [20, 27–32].

The dosage balance model contends that, for genes that are in stoichiometric balance, any perturbation of their relative ratios is deleterious [26, 32, 33]. Under this model, sets of dosage-balanced genes can only change copy number in concert or not at all. There are multiple lines of evidence for this model, including: the copy number of different components of the ribosome co-vary to retain stoichiometric balance [34]; in some cases an artificially induced overexpression phenotype can be rescued by the overexpression of an interacting partner [35]; and genes whose products form protein complexes are not normally duplicated [36].

## Dosage-sensitive genes are duplicated by polyploidy or not at all

The knowledge that some genes in the genome are sensitive to copy number changes, should they occur, suggests a model where these genes have persistent sensitivity to evolutionary dosage changes, whereas other genes have no such sensitivity. The tendency of a gene to be duplicated or not is referred to as ‘duplicability’. The observation that duplicability is a relatively stable property of a gene, with some genes consistently found as singletons and others repeatedly independently duplicated across distant lineages [37], supports the existence of ancient and persistent dosage constraints on genes. However, it is useful to remember that these dosage constraints are not a limit on the absolute number of copies of a gene, but on the ratio of a gene product to other components of the cell, either in terms of overall concentration or in terms of specific interacting partners [26, 27, 38–40]. So, the non-duplicability of dosage-sensitive genes relates to one-by-one duplication or loss and not to concerted events. Indeed, if a given set of dosage-balanced genes were to be linked on a chromosome, then a single segmental duplication including all of them may not be deleterious [41].

By definition, even genes that are not normally ‘duplicable’ are duplicated by whole genome duplication (WGD; or polyploidy). This event creates no deleterious imbalance, because, even though all of the genes are duplicated, the ratios remain unchanged. During the subsequent period of extensive gene loss that has followed every known polyploidy event [42] deletion of some, but not all, of a set of dosage-balanced genes would be deleterious, being imbalanced. This leads to the prediction that such genes should be preferentially retained through this period of purging of paralogs [43]. Consistent with expectations based on dosage sensitivity, we and others have observed that genes that are not generally duplicable by small-scale duplication (SSD) are in fact disproportionately retained after polyploidy [44–48]. The patterns of general duplicability by SSD and of retention after WGD are so contrasting as to result in almost completely non-overlapping groups of genes.

The dosage balance model prediction that WGD paralogs (termed ‘ohnologs’ [42, 49]) should be enriched for dosage-sensitive genes is supported by the observation that most ohnologs are unduplicated even in lineages that diverged prior to the WGD event and do not experience subsequent duplications except if by another WGD event [44, 47]. This duplication constraint extends into recent population polymorphism: we found that ohnologs are rarely observed in CNVs in healthy individuals, whereas genes that are frequently copied by SSD also commonly have benign CNVs [47]. In other words, the general trend is that genes that can be individually duplicated, are; by contrast dosage-sensitive genes are duplicated by polyploidy, or not at all.

## CNVs and dosage sensitivity

The existence of CNVs has been long known, but since they were recognised as a significant category of genetic variation 13 years ago [50] our understanding of how they are generated and their phenotypic consequences has grown. Like any kind of genetic variation, the global distribution of CNVs is determined by demography and selection [51–53]. Many CNVs are selectively neutral [2], sometimes even when present as homozygous deletions [54]. Instances of positive selection include adaptations to diet [55] and pathogen resistance [56]. However, a significant number of CNVs are deleterious and associated with disorders [57, 58], including developmental delay [6], hearing loss [59], heart disease [7] and neuropsychiatric conditions [60, 61].

**Fig. 2.**
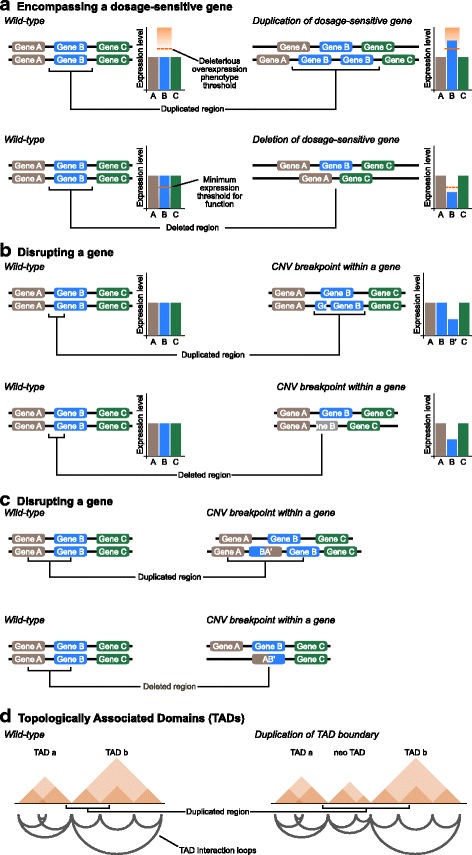
Multiple different ways in which a CNV can have a pathogenic effect. **a** CNVs cause duplication and/or deletion of the enclosed genes. If one or more of those genes is dosage-sensitive then there will be a consequent phenotype, usually deleterious. **b**, **c** Alternatively, CNVs with breakpoints within a gene disrupt the gene by truncation (**b**) or formation of chimeras (**c**). Gene truncation will usually result in loss of function, but may alternatively result in a gain of function, dominant negative effect. Chimeric genes have unpredictable effects, and may be pathogenic. **d** Topologically associating domains (TADs) are structural units in the three-dimensional organisation of the genome and play a large role in mediating gene–enhancer interactions and other aspects of gene expression regulation. TADs are isolated from each other by TAD boundaries, which are determined by protein binding sites. CNVs encompassing TAD boundaries create new TADs. These can result in rewiring of gene enhancer interactions including the isolation of a gene from its regulator or the placement of a gene under the regulation of an inappropriate enhancer. Disruption of TADs has been associated with human disease [64–66, 68]

Obviously, any CNV changes the copy number of genes contained within its breakpoints, but it is not necessarily the case that the phenotype of any given CNV is due to gene dosage changes as the CNV will also potentially disrupt genes, uncouple genes from their regulatory sequences, or alter chromosome three-dimensional organisation [62–68] (Fig. 2). Nonetheless, dosage sensitivity of the encompassed genes is the most popular hypothesis to explain pathogenic CNVs, with multiple examples known [69]. This view was supported by an evolutionary analysis of gene duplication and loss across mammals where we found that genes in pathogenic CNVs have much more conserved copy number than genes observed in CNVs in healthy individuals [48]. This observation is uniquely explained by copy number constraints on the enclosed genes rather than any other model of CNV pathogenicity.

## Dosage sensitivity and genome evolution

Not surprisingly, dosage-sensitive genes have also played an enormous role in the evolution of gene content and gene expression of sex chromosomes. Not only did they precipitate the evolution of elaborate dosage compensation mechanisms [70–72], but they also have shaped gene content through both purifying selection and relocation of dosage-sensitive genes to autosomes [73–77]. For other dosage-sensitive genes that did not relocate to autosomes, especially members of large protein complexes, we and others have shown that the expression of sex-chromosome-linked genes and their autosomal interacting partners has evolved so as to maintain stoichiometric balance [78, 79].

Because dosage-sensitive genes are refractory to duplication events, and because duplications are often long, encompassing multiple genes [80], the simple presence of dosage-sensitive genes has incidental effects on neighbouring genes. The likelihood that a duplication of a given gene also includes a dosage-sensitive gene should decrease with the physical distance between them. Using ohnologs as a proxy for dosage-sensitive genes, we found evidence in support of this model, as the closer a gene is to an ohnolog the less likely it is to be duplicated [81]. This effect is sufficiently strong to create SSD and CNV deserts in the human genome [81].

One of the principal mechanisms of generation of CNVs is by non-allelic homologous recombination (NAHR; recombination events between different loci with high sequence similarity) [82]. Approximately 10% of the human genome is subject to recurrent CNVs due to the existence of NAHR hotspots [83]. These hotspots are created by the presence of segmental duplications (low-copy repeat sequences) that are at least 95% identical at the DNA level, at least 10 kb long, and located between 0.05 and 10 Mb apart [84]. At least 2129 known recurrent pathogenic CNVs occur at NAHR hotspots [85] and in at least two cases new human disease-associated NAHR hotspots were created by recent lineage-specific segmental duplication events [17, 86]. In both cases there is an as yet unproven claim that the duplication event was itself adaptive, thus compensating for the risk of disease in offspring [86, 87]. It remains unknown how the propensity of segmental duplications flanking dosage-sensitive genes to generate pathogenic CNVs has impacted upon genome evolution. One might expect purifying selection to destroy the NAHR hotspots around dosage-sensitive genes, perhaps by genome rearrangement events that eliminate the proximity of the segmental duplications.

## Evolutionary patterns are an informative trait to identify human disease genes

One of the recurrent pathogenic CNVs in humans occurs at 22q11, resulting in 22q11 deletion syndrome, which has a variable phenotype including heart defects, developmental disorders and schizophrenia [14]. Similarly, recurrent NAHR at 16p11.2 generates duplication and deletion CNVs, both of which are pathogenic, but which have different, ‘mirrored’ phenotypes impacting metabolism, developmental delay and neuropsychiatric traits [88, 89]. At both 22q11 and 16p11.2 there is a large CNV but also a smaller, ‘critical’ CNV with the same phenotype. As such, in each case the phenotype is considered to be due to dosage sensitivity of some of the genes within the smaller regions. However, even within the critical regions the number of genes is still quite large, numbering 28 and 26 for 22q11 and 16p11.2, respectively. An important challenge is to identify the most likely candidate genes for the phenotype from among these.

**Fig. 3.**
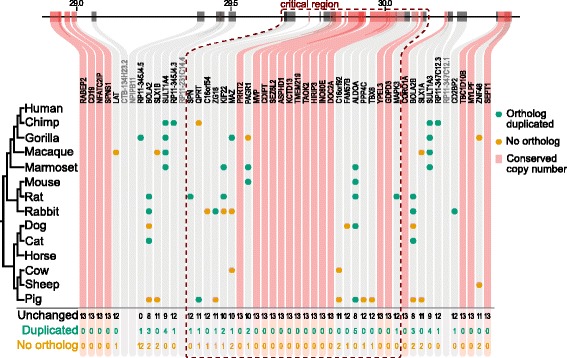
Evolutionary conservation of copy number of genes in the 16p11.2 recurrent CNV region. The genes in the 16p11.2 region are illustrated across the top, with Mbp co-ordinates indicated above and gene names below. The critical region (*dashed outline*) indicates a smaller CNV that exhibits the same phenotype as the larger CNV and so is considered sufficient for the syndrome. For each of the mammals, if the ortholog is duplicated it is represented by a *green dot*, and if not found (presumed deleted) it is represented by an *orange dot*. Otherwise the copy number is unchanged with respect to human. Where a given gene has 1:1 orthologs across all 13 mammals tested this is indicated by a *red vertical stripe*. Genes in the region that were not amenable to this analysis are indicated by *greyed-out names*. Copy number conservation data are from [48]. *BOLA2, SLX1* and *SULT1A* are part of a human-specific duplication with paralogs present on both flanks of the critical region and which increased the susceptibility to NAHR [86]

Evolutionary analysis provides a powerful method for pinpointing the dosage-sensitive genes within these and other CNV regions. Our detailed inspection of the evolutionary copy number conservation of the genes in the 22q11 deletion syndome region revealed that orthologs of 16 out of the 28 genes are present (never lost) across 13 mammalian genomes analysed [48]. Similarly, in the 16q11.2 region, 13 out of the 26 genes in the critical region have completely conserved copy number (1:1 orthologs) in all mammalian genomes analysed (Fig. 3). These completely conserved genes fit the profile expected of dosage-sensitive genes, and as such are attractive candidate genes for the syndrome.

Trisomies are chromosomal abnormalities that are at least conceptually comparable to large CNVs. Trisomy 21, which results in Down’s syndrome, is the most common human trisomy, occurring in approximately 1 in 700 live births [90]. The next most common human trisomies, 18 and 13, can survive briefly post-birth and result in Edward's syndrome and Patau syndrome, respectively. Other trisomies occur but are inviable [91]. The high frequency of trisomy 21 reflects the fact that it results in a relatively mild syndrome.

The pathogenic effects of the trisomy are likely to be due to a combination of the effects of the specific genes on the chromosome [47, 90] and general expression dysregulation [92]. In yeast the phenotype of an aneuploidy is largely independent of the identity of the chromosome [93], suggestive of a general disruption, not specific to the biochemical function of particular genes. Why human trisomy 21, 18 and 13 in particular are unusually viable is probably related to the low number of genes encoded on each chromosome. However, as with pathogenic CNVs, it is likely that only a subset of these are dosage sensitive. There is a very interesting correlation with the number of ohnologs (here again, a proxy for dosage-sensitive genes) and trisomy severity. Human chromosome 21 has the most common (least severe) trisomy and the smallest number of ohnologs. Similarly, chromosomes 18 and 13 have the next smallest numbers of ohnologs, and the next highest trisomy incidences, respectively (Table 1). By contrast, none of the mouse trisomies is viable (only mouse trisomy 19 survives 1–4 weeks post-birth [94]). However, there is a similar correlation between the number of ohnologs and the gestational survival of trisomies (Table 2). At least for human chromosome 21 we found that this number of ohnologs is surprisingly small even given its small number of genes [47]. Three-quarters of previously reported Down’s syndrome candidate genes were independently discovered by this evolutionary analysis, but other genes were also identified as under evolutionary constraint that were not previously recognised to have an association with the syndrome. Thus, we argued that these genes, distinguished by their characteristic pattern of evolutionary copy number conservation, are interesting candidate genes for Down’s syndrome [47]. Though aspects of the syndromes are clearly related to the specific genes on the chromosome, the correlation with number of genes encoded and perhaps specifically with the number of ohnologs suggests a general relationship with the extent of the burden of dosage-sensitive genes.

**Table 1:** Human autosomes with common trisomies, in ascending order of number of ohnologs

Human chromosome number	Number of dosage-balanced ohnologs^a^	Trisomy syndrome	Frequency (number of live births)^b^
21	61	Down's	1/700
18	98	Edward's	1/5000
13	138	Patau	1/16,000

^a^Identified from gene trees in Ensembl v86 [109] as genes that duplicated at the base of the vertebrate tree with no subsequent SSD

^b^Frequency data from [90] and ghr.nlm.nih.gov/trisomy-18; ghr.nlm.nih.gov/trisomy-13

**Table 2:** Mouse autosomes in ascending order of number of ohnologs

*Mus musculus* chromsome number	Number of dosage-balanced ohnologs^a^	Trisomy survival^b^
18	194	To term
16	201	14 days post-fertilisation—term
12	218	12–17 days post-fertilisation
13	232	13 days post-fertilisation—term
19	235	1–4 weeks post birth

^a^Identified from gene trees in Ensembl v86 [109] as genes that duplicated at the base of the vertebrate tree with no subsequent SSD

^b^Mouse trisomy survival data obtained from [94]. No unlisted trisomies survive past 19 days post-fertilisation

## Gene expression burden as a possible explanation for duplication phenotypes

As mentioned earlier, we and others found that genes that are successfully duplicated by SSD are usually not retained after WGD, and vice versa [46, 47, 81, 95, 96]. Curiously, these contrasts also carry over into the differences in gene expression level and coding sequence length. Whereas in human the median expression for SSD paralogs is 13.4 RPKM (reads per kilobase of transcript per million mapped reads) and the median CDS length is 1206 nucleotides, these values are much higher for ohnologs (23.7 RPKM and 1557 nucleotides, respectively). A similar pattern is seen in paramecium [97].

These contrasting patterns of expression for SSD paralogs and ohnologs could be explained if there are very different consequences of duplicating one (or a few) highly expressed gene(s) by SSD, compared to balanced duplication of all genes simultaneously by WGD. These different consequences might arise not only because some genes have dosage constraints (such as the requirement to maintain balanced ratios between specific gene products) and thus cannot be duplicated individually [38, 47, 98], but also because an extra copy of a highly expressed gene may be costly in terms of cellular resources [99–101].

This latter idea is consistent with observations from yeast where it was shown that overexpression of highly expressed genes has a greater negative effect than of less highly expressed genes [35]. The reported experiments linked the deleterious phenotype to the protein burden rather than the protein biochemical function (the phenotype was recapitulated when the protein sequence was replaced by green fluorescent protein (GFP)) but did not dissect the nature of that burden.

WGD defies this cost, as can be seen plainly in the readiness with which plant genome ploidy increases [102], an extreme example being oilseed rape which has a 72-fold increase since the origin of angiosperms owing to multiple WGD events [103]. This makes intuitive sense if the WGD increases and draws down cellular resources evenly, with no net difference compared to the pre-WGD genome. It has previously been shown that the greater the proportionate increase in copy number, the greater the phenotypic consequence; that is, adding one extra copy to a haploid is more dramatic than adding one extra copy to a diploid [30]. Thus, the greater the number of copies of a given gene, the lesser the impact of one more copy. One could therefore predict that the effects of SSD in a genome with a history of multiple WGD events, like that of oilseed rape, would be much reduced compared to an outgroup.

## Is gene expression a zero-sum game?

The idea of a cost associated with gene expression has rich theoretical support and experimental evidence [99–101, 104–106]. Furthermore, highly expressed genes are expected to be particularly dosage sensitive [35, 97]. Protein expression is a significant fraction of a cell's energy budget [99, 100, 107]. The cost of expression can be expanded to include a model where overexpression of one locus is not merely a waste, but actually sequesters cellular resources away from other genes. Clearly the number of RNA polymerases available for transcription and the number of ribosomes available for translation are both finite, but are they sometimes limiting? Experiments in *Escherichia coli* found the rather surprising result that overexpression of one locus could lead to a depletion of ribosomes [104]. Other experiments showed that the cost of overexpression is not due to protein biochemical function or amino acid usage, but can be fully explained by the cost of the process of gene expression [101]. Rather than simply requiring resources, overexpression of some genes should naturally titrate out polymerases, ribosomes and other cellular resources so that they become unavailable for other genes. In other words, a duplication of a ‘greedy’, highly expressed gene might exert a deleterious effect by indirectly lowering the expression of other genes. This model views gene expression as a ‘zero sum game’, where an increase in one gene may cause decreased output from another. This is consistent with a recent model of human disease dubbed the ‘omnigenic’ model, where regulatory changes to any gene expressed in a disease-relevant tissue may contribute to disease whether or not there is a direct mechanistic link to the disease phenotype [108]. The authors of the omnigenic model suggest that gene regulatory networks are so interconnected as to allow changes in any expressed gene to affect any other.

Under the ‘zero sum’ model the barriers to duplication, that is, the costs of duplication, will sometimes lie not in the biochemical function of the gene that has been duplicated (though this is undoubtedly the case in many instances) but in the cost of the process of gene expression in terms of both energy (the cell spends seven ATPs for every amino acid of a protein) and the sequestration of the cell machinery such as polymerases and ribosomes. If RNA polymerases and ribosomes are limiting factors in the amount of gene expression possible from a given cell, then titration of these macromolecules by the doubling of a long, highly expressed gene would have knock-on consequences for the expression of other highly expressed genes, which may become reduced to pathogenic levels. Notably, this cost would not be incurred in WGD because all components of the system, including expression machinery, are duplicated equally, and all ratios remain constant.

## Outlook – Gene expression as a keystone to understanding copy number constraints?

This view of gene dosage sensitivity may sit alongside other more well-established modes of dosage sensitivity [30]; however, it is currently speculative. In particular one must query whether duplication of one highly expressed locus would have sufficient impact on cellular resources when expressed at physiological levels to have any effect. The costs ought to matter more in rapidly proliferating cells, which may limit this to microbial organisms [100], but they may also be relevant for quickly growing tissues. This is an interesting area to explore as it has the potential to explain some common trends in evolutionary gene duplication by different mechanisms and link this propensity to disease phenotypes of human CNVs and trisomies.
